# Assessing attitudes to ED-based HIV testing: Development of a short-structured survey instrument

**DOI:** 10.1371/journal.pone.0252372

**Published:** 2021-05-27

**Authors:** Aditi Rao, Emily M. Nagourney, Victoria H. Chen, Sarah Hill, Eili Y. Klein, Madeleine Whalen, Thomas C. Quinn, Bhakti Hansoti

**Affiliations:** 1 Department of International Health, Johns Hopkins Bloomberg School of Public Health, Baltimore, MD, United States of America; 2 Krieger School of Arts and Sciences, Johns Hopkins University, Baltimore, MD, United States of America; 3 Department of Emergency Medicine, Johns Hopkins University School of Medicine, Baltimore, MD, United States of America; 4 Department of Medicine, Johns Hopkins University School of Medicine, Baltimore, MD, United States of America; 5 Division of Intramural Research, National Institutes of Allergy and Infectious Diseases, NIH, Bethesda, MD, United States of America; Hofstra University, UNITED STATES

## Abstract

**Introduction:**

Emergency Department (ED)-based HIV counseling and testing (HCT) has had a significant impact on improving rates of HIV diagnosis and linkage to care. Unfortunately, expansion of this strategy to low- and middle-income countries has been limited. Successful implementation of ED-based HCT is dependent on patient and provider acceptance of the intervention, and their attitudes and pre-existing biases towards the disease. This study sought to develop validated survey instruments to assess attitudes towards ED-based HCT.

**Methods:**

This cross-sectional study surveyed patients and providers in three EDs in the Eastern Cape province, South Africa. A convenience sample of patients and providers in the ED were surveyed. Exploratory factor analysis was conducted using questions on attitudes to HIV testing to develop validated survey instruments. An ANOVA test assessed variance in attitudes towards HCT based on demographic variables collected.

**Results:**

A total of 104 patient and 132 provider surveys were completed. Exploratory factor analysis resulted in a 17- and 7-question attitudes survey for patients and providers, respectively. Overall, 92.3% of patients and 70.7% of providers supported ED-based HCT, however, both groups displayed only mildly positive attitudes. Questions representing ‘confidentiality’ and ‘stigma around HIV testing’ had the least positive influence on patients’ overall attitudes. Questions representing ‘comfort with HIV testing’ had the least positive influence on providers’ overall attitudes.

**Conclusion:**

Our study demonstrated ED patients and providers are generally supportive of ED-based HCT. A validated survey instrument was able to provide a standardized approach to identify barriers to HCT implementation in an ED setting, across contexts. For successful implementation, behavioral interventions must focus on strengthening patient beliefs around confidentiality and the consent process, and providers’ comfort levels with providing HIV testing services in the ED.

## Introduction

Emergency Departments (ED) are a potential venue to expand access to HIV counselling and testing (HCT) services to otherwise missed populations [1–3]. In the United States, ED-based HCT has had a significant impact on improving the rates of HIV diagnosis and subsequent linkage to care [4–6]. Unfortunately, the expansion of this strategy to low- and middle- income countries (LMIC) has been limited [7]. Implementing ED-based HCT will help improve access to HIV treatment and care for populations who do not regularly interact with the healthcare system through common and more established channels, such as the private health sector [2, 8]. South Africa has the highest prevalence of HIV in the world [9, 10], with much of its HCT resources predominantly directed to primary health care centers and antenatal clinics, neglecting other high-volume venues, such as EDs [2, 11]. Wherein, ED-based HCT is not routinely implemented due to lack of standardized training, competing clinical priorities, and in-sufficient resource allocation [3, 12–14]. However, a significant proportion of the population in South Africa relies on EDs as the sole point of contact with the healthcare system [15], as such ED-based HCT in South Africa has the potential to extend necessary HIV services to populations not captured through other testing venues. Furthermore, recent studies demonstrate a high prevalence of HIV in the ED (28%), of which almost a third of patients were unaware of their status [16].

Successful implementation of ED-based HIV testing interventions, rest on understanding the feasibility and organizational readiness for adoption [17]. Understanding patient and provider attitudes to ED-based testing can inform organizational readiness for change and, thus inform the successful implementation of HCT services [18, 19]. While there is anecdotal understanding of local attitudes towards ED-based testing programs in LMICs, there is no standardized, validated assessment tool currently available. Attitudes, defined as a complex mental state involving beliefs, feelings, values, and dispositions to act in certain ways [20], are essential to determine in order to suitably evaluate acceptability, appropriateness, and feasibility of a complex intervention. Several survey tools already exist to capture patient and provider attitudes to HIV testing programs, however, none of them have been validated for use outside of the US. In this study we propose to use existing survey instruments within our context, to develop a short-validated survey instrument assessing patient and provider attitudes towards ED-based HCT across contexts.

## Methods

### Study design and setting

The observational survey study was conducted between September 2016 and September 2017 in three hospitals in the Eastern Cape province of South Africa: Frere Hospital (FH), Nelson Mandela Academic Hospital (NMAH), and Mthatha Regional Hospital (MRH).

The Eastern Cape province is historically under-resourced and experiences the second highest prevalence of HIV infection in the country [9]. It is the third most populous region in South Africa, constituting 12.6% of the country’s population and faces a disproportionate burden of acute injuries and illnesses with high rates of HIV, sexually transmitted infections (STI), and tuberculosis (TB) [9, 15]. The Eastern Cape remains one of the poorest provinces in the country, and has been identified as a key priority area for HIV research and capacity building in South Africa [21].

Frere Hospital (FH) is a provincial, government-funded facility located in East London. Nelson Mandela Academic Hospital (NMAH) and Mthatha Regional Hospital (MRH) are located in Mthatha and affiliated with Walter Sisulu University. NMAH is a large tertiary-care referral center with 24-hour trauma services, and MRH is a district-level facility that provides services to walk-in patients as well as referrals from clinics. All three hospitals serve 100–150 patients per day from their surrounding 100km catchment area. The sites are low-resource and are not equipped with an Electronic Medical Record (EMR) system, patient tracking systems, or standardized triage processes. Furthermore, there are no Emergency Medicine specialized providers at the three sites.

### Study population and recruitment

A convenience sample of patients and providers were enrolled from the emergency department. We attempted to survey every 10^th^ patient who presented for care to the ED, aged 18 years or older and clinically stable [22]. Patients were approached in the ED waiting room so as not to interfere with patient care. Providers were informed of the study through announcements made during the morning and evening shift change, we attempted to capture all providers working in the ED. Providers were approached at the start or end of their shift, or during breaks. Across the health facilities approximately 60% of providers agreed to participate. Surveys were conducted in a private setting to ensure as much confidentiality as possible. All data were recorded anonymously and no patient identifying data were collected.

### Survey instrument

The study team developed two surveys to assess patient and provider attitudes towards ED-based HCT using a deductive approach. Experts in the study team evaluated the survey for content validity. The patient survey had a total of 83 questions, which included 18 demographic questions (i.e., age, gender, race, level of education etc.), 13 questions assessing ‘risk behaviors’, nine questions assessing ‘HIV knowledge’, and 43 questions assessing ‘patient attitudes to HIV testing’ (S1 Table). The provider survey had a total of 53 questions, which included seven demographic questions (i.e., age, gender, race, current position, etc.), 34 questions assessing ‘HIV stigma’, and 12 questions assessing ‘provider attitudes to HIV testing’ (S2 Table). Questions assessing ‘risk behaviors’ and ‘HIV knowledge’ for patients, and ‘HIV stigma’ for providers were pulled from previously validated questionnaires and not further modified [19, 23]. Questions assessing patient and provider ‘attitudes to HIV testing’ were pooled from previously published instruments [6, 24–28] and underwent exploratory factor analysis resulting in a distilled pool of questions to capture the maximum possible variation in attitudes in the fewest number of questions.

### Data collection

Study staff completed patient and provider surveys over a period of six weeks at each of the three hospitals. The surveys were conducted in English or Xhosa depending on patient or provider preference and proficiency. The survey included a brief introduction to the study and asked for verbal consent prior to recording any data. Questions were mostly read out loud followed by answer options when applicable. Questions were repeated as needed, but not interpreted for the participant. Some providers preferred to read and input their answers into the tablet themselves. Data were collected using an electronic handheld mobile tablet, using the Qualtrics application (Qualtrics, Provo, UT). At the end of the study all responses were exported into Microsoft Excel v.16.9 (Microsoft Inc.), then imported into Stata v.14 (StataCorp, Tx) for analysis.

Demographic data were recorded using pre-defined categories, while questions on HIV knowledge and HIV risk factors were binary with ‘true’ or ‘false’ responses. Scoring for attitude questions was performed on a 5-point Likert scale with 1 being ‘strongly disagree’ and 5 being ‘strongly agree’. Negatively worded questions were reversed in numeric value, so the number 5 consistently reflected a positive attitude.

### Survey validation

We conducted exploratory factor analysis using the attitude survey questions (43 questions for patients and 12 questions for providers) to develop validated survey instruments in this context. Factorability of the survey questions was determined by inspecting the correlation matrix (correlations > 0.4), the Kaiser-Meyer-Olkin measure of sampling adequacy (KMO > 0.6), and Bartlett’s test of sphericity (p < 0.05). Survey questions were then subjected to factor analysis with an oblique rotation (oblimin), producing as simple a structure as possible while permitting correlations among factors. Factors were retained based on the Scree test [29], Kaiser’s rule [30], and parallel analysis [31]. Cronbach’s alpha coefficient was calculated to test for reliability and internal consistency within and between factors. Using a regression method, factor scores were calculated for all factors for each participant.

Factor scores were standardized and compared across demographic categories using one-way ANOVA to assess if there were any trends or variances in attitudes towards HCT. These variables include ‘gender’, ‘race’, ‘age’, and ‘highest level of education’, in addition to ‘employment status’ and ‘previously tested for HIV’ for patients, and ‘current position’ and ‘years of practice’ for providers. A p-value of ≤0.05 was regarded as statistically significant. A pooled analysis of data collected from the three sites is presented; no significant differences in characteristics of respondents as well as attitudes towards ED-based HCT were observed between the sites.

### Outcomes

The primary outcome of interest was patient and provider attitudes towards ED-based HCT. The secondary outcome of interest was whether patient and provider attitudes varied by demographic variables.

### Ethical considerations

This study was approved by the Johns Hopkins University School of Medicine Institutional Review Board, the University of Cape Town Human Research Ethics Committee, and the Walter Sisulu University Human Research Ethics Committee. Verbal consent was obtained from all participants who enrolled in the study.

## Results

A total of 104 patient and 132 provider surveys were completed (Table 1). Among patient responses, a majority were female (n = 54, 51.9%), between the ages of 30 and 39 years (n = 36, 34.6%), of African race (n = 86, 80.8%), had completed some or all of high school (n = 79, 75.9%), were unemployed (n = 65, 62.5%), and had previously been tested for HIV (n = 67, 64.4%). Among provider responses a majority were female (n = 93, 69.9%), between the ages of 20 and 29 years (n = 49, 36.8%), of African race (n = 107, 80.4%), and had been practicing medicine for 0 to 4 years (n = 48, 36.1%). Further, provider responses indicate 45 (33.8%) were physicians, 52 (39.1%) were registered nurses, 19 (14.3%) were nursing assistants/practitioners, and 16 (12.8%) were case managers.

**Table 1:** Characteristics of respondents by site.

Variable	Patient Responses (%)^†^				Provider Responses (%)^†^			
	Frere n = 46	NMAH n = 27	MRH n = 31	Total n = 104	Frere n = 53	NMAH n = 50	MRH n = 30	Total n = 133
**Age**								
< 20	2 (4.35%)	2 (11.11%)	4 (12.90%)	8 (7.69%)	0 (0%)	1 (2.0%)	0 (0.0%)	1 (0.75%)
20–29	9 (19.57%)	6 (22.22%)	11 (35.48%)	26 (25.0%)	14 (26.42%)	17 (34.0%)	18 (60.0%)	49 (36.84%)
30–39	19 (19.57%)	10 (37.04%)	7 (22.58%)	36 (34.62%)	10 (18.89%)	15 (30.0%)	6 (20.0%)	31 (23.31%)
≥ 40	16 (34.78%)	9 (33.33%)	9 (29.03%)	34 (32.69%)	27 (50.94%)	17 (34.0%)	6 (20.0%)	50 (37.59%)
**Sex**								
Males	20 (43.48%)	14 (51.85%)	15 (48.39%)	49 (47.12%)	11 (20.75%)	19 (38.0%)	8 (26.7%)	38 (28.57%)
Females	26 (56.52%)	13 (48.15%)	15 (48.39%)	54 (51.92%)	40 (75.47%)	31 (62.0%)	22 (73.3%)	93 (69.92%)
**Race**								
African	35 (76.09%)	23 (85.19%)	28 (90.32%)	86 (82.69%)	34 (64.15%)	46 (92.0%)	27 (90.0%)	107 (80.45%)
Coloured	5 (10.87%)	0 (0.0%)	0 (0.0%)	5 (4.81%)	12 (22.64%)	2 (4.0%)	3 (10.0%)	17 (12.78%)
White	6 (13.04%)	4 (14.81%)	3 (9.68%)	13 (12.50%)	6 (11.32%)	2 (4.0%)	0 (0.0%)	8 (6.02%)
**Highest level of education**								
< High school	5 (10.87%)	5 (18.52%)	11 (35.45%)	21 (20.19%)	0 (0.0%)	0 (0.0%)	0 (0.0%)	0 (0.0%)
Some or all of high school	34 (73.91%)	22 (81.48%)	19 (61.29%)	79 (75.96%)	14 (26.42%)	1 (2.0%)	1 (3.33%)	16 (12.03%)
≥ Bachelor’s degree	3 (6.98%)	0 (0.0%)	1 (3.23%)	4 (3.85%)	38 (71.70%)	49 (98.0%)	29 (96.67%)	116 (87.22%)
**Employment**								
Yes	19 (41.30%)	11 (40.74%)	9 (29.03%)	39 (37.5)	-	-	-	-
No	27 (58.70%)	16 (59.26%)	22 (70.97%)	65 (62.5%)	-	-	-	-
**Position**								
Physicians	-	-	-	-	14 (26.42%)	17 (34.0%)	14 (46.67%)	45 (33.83%)
Registered nurse	-	-	-	-	29 (54.72%)	16 (32.0%)	7 (23.33%)	52 (39.10%)
Nursing assistants / practitioners	-	-	-	-	5 (9.43%)	10 (20.0%)	4 (13.33%)	19 (14.29%)
Case managers	-	-	-	-	4 (7.55%)	7 (14.0%)	5 (16.67%)	16 (12.03%)
**Years of practice**								
0–4 years	-	-	-	-	8 (15.09%)	21 (42.0%)	19 (63.3%)	48 (36.09%)
5–9 years	-	-	-	-	22 (41.51%)	16 (32.0%)	8 (26.66%)	46 (34.59%)
>10 years	-	-	-	-	20 (37.74%)	13 (26.0%)	3 (30.0%)	36 (27.07%)

† Data were missing for some variables, therefore numbers do not always add to the total.

### Patient attitudes to testing

Overall, 92.3% (n = 96) patients supported ED-based HCT. A majority of patients (80.8%, n = 84) agreed that if offered, they would assent to testing, and 72.1% (n = 75) patients reported wanting to learn more about HIV. However, 71.2% (n = 74) patients also stated that if ED-based testing were implemented, they would prefer an HIV counsellor to carry out testing and disclose results, instead of a physician or nurse. Patients’ knowledge on HIV infection, transmission and management was generally good, wherein the median score from the nine questions on HIV knowledge was 8 [CI: 7–9].

#### Factor analysis and scores

Survey validation using exploratory factor analysis resulted in a 17-question instrument (from the original 36 survey questions) with seven factors, accounting for 74.6% of the total variance of the sample. Questions with a factor loading less than 0.4 and those cross-loading on more than one factor were omitted. Factors with only one question loading on to it were also omitted.

Table 2 shows the mean scores for the 17 survey questions used to evaluate patient attitudes. A mean score of 3 represents an overall neutral attitude, while a mean score more or less than 3 represents an overall positive or negative attitude, respectively. Patients demonstrated an overall mean score of 3.27, indicating a neutral attitude towards ED-based HCT. For example, question 5 ‘*I want to learn more about HIV*’ has a mean score of 3.48, demonstrating patient willingness to engage and learn more. Question 10 ‘*The ED should offer HIV testing*’ has a mean score of 4.43, showing a strongly positive attitude towards having ED-based HCT. On the other hand, question 7 ‘*The ER and hospital can test you for HIV without asking for your consent’* has a mean score of 2.77, indicating the presence of concern or mistrust of clinical facilities in this population.

**Table 2:** Mean patient and provider scores by question.

Patient Attitude Questions	Mean Score*
1. The results of a negative HIV test can be disclosed where beds are separated only by curtains.	3.95
2. The results of a positive HIV test can be disclosed where beds are separated only by curtains.	2.84
3. Patients should be provided with counselling prior to the offering of testing.	3.87
4. Patients should be required to given consent prior to testing.	3.99
5. I want to learn more about HIV.	3.48
6. I want to learn ways to avoid getting HIV.	3.71
7. The ED and hospital can test you for HIV without asking for your consent.	2.77
8. People assume that everyone who is tested for HIV is infected with HIV.	2.89
9. I trust the HIV testing counselors and nurses to keep my information private and confidential.	3.45
10. The ED should offer HIV testing.	4.43
11. It doesn’t matter who tells me my HIV result	3.28
12. I think that the hospital already tests every patient for HIV without telling them about it.	3.62
13. If I have been in the hospital, and no one told me I had AIDS or HIV, then I am negative.	3.26
14. My parents would be upset if they knew I was planning to get tested for HIV.	2.23
15. My friends would support my decision to get an HIV test.	2.57
16. I would not want anyone to know if I decided to test for HIV.	2.91
17. Anyone who is tested for HIV is disgusting.	3.38
**Total (all 17 questions)**	**3.27**
**Provider Attitude Questions**	Mean Score*
1. The ED should offer HIV testing.	3.68
2. The ED should offer HIV testing to ALL patients.	3.64
3. The ED should offer HIV testing to high-risk patients only.	2.49
4. ED patients will benefit from knowledge of their HIV status.	4.36
5. Offering HIV testing will take too much time and will interfere with my job duties.	3.00
6. I am afraid that if we ask patients about HIV testing, they will be offended or upset.	3.38
7. I am comfortable disclosing the results of a positive HIV test to a patient.	3.50
**Total (all 7 questions)**	**3.44**

* 1 = least positive attitude, 5 = most positive attitude

The seven patient factors were identified as representing, ‘confidentiality’, ‘counseling & consent’, ‘openness to HIV knowledge’, ‘stigma around HIV testing’, ‘ED-based testing’, ‘social support’, and ‘stigma around HIV infection’ (attitudes questions included in each factor can be viewed in S3 Table). Mean scores for each factor in Table 3 signify the baseline attitudes of patients towards ED-based HCT. Overall, patients showed a positive attitude for each factor. Patient factor scores show that ‘confidentiality’ and ‘stigma around HIV testing’ have the least positive influence on patients’ attitudes towards ED-based HCT.

**Table 3:** Mean patient and provider scores by factor (re-scaled to mean of 3.27).

**Patient Factors**	Mean Score*
1. Confidentiality	2.99
2. Counseling and consent	4.71
3. Openness to HIV knowledge	3.60
4. Stigma around HIV testing	3.03
5. ED-based HIV testing	3.70
6. Social support	3.36
7. Stigma around HIV infection	3.12
**Provider Factors**	Mean Score*
1. Benefits of HIV testing	3.44
2. Comfort with providing HIV testing	3.43

* 1 = least positive attitude, 5 = most positive attitude

### Provider attitudes to testing

Overall, 70.7% (n = 94) of providers supported ED-based HCT, and 97.0% (n = 129) of providers felt it is important that individuals are aware of their HIV status. However, 52.6% (n = 70) of providers agreed that ED-based HCT will take up too much time and interfere with their job duties, and 76.7% (n = 102) of providers reported the lack of adequate support staff available in the ED for HCT. Additionally, 88.7% (n = 118) of providers agreed that if ED-based HCT were implemented, they would prefer an HIV counsellor to carry out testing and disclose results to patients. Providers reported varying degrees of stigma around HIV infection, transmission and management, wherein 69.2% (n = 92) of providers believed that HIV positive patients engage in risky activities despite knowing the risks, but only 17.3% (n = 23) of providers agreed that HIV positive patients present a threat to their health. 18.8% (n = 25) of the providers surveyed reported the belief that they have the right to refuse care to HIV positive patients.

#### Factor analysis and scores

Survey validation using exploratory factor analysis resulted in a 7-question instrument (from the original 12 survey questions) with two factors, accounting for 95.1% of the total variance of the sample. Questions with a factor loading less than 0.4 and those cross-loading on more than one factor were omitted.

Table 2 shows the mean scores on the 7-survey questions used to evaluate provider attitudes. Providers demonstrated an overall mean score of 3.34, suggesting that providers have a generally neutral attitude towards ED-based HCT. For example, question 2 *‘The emergency department should offer HIV testing to ALL patients’*, has a mean score of 3.68, and question 5 *‘Emergency department patients will benefit from knowledge of their HIV status’*, has a mean score of 4.36, demonstrating that providers see utility in an ED-based testing strategy. On the other hand, question 4 *‘Offering HIV testing will take too much time and will interfere with my job duties’* has a mean score of 2.86, which demonstrates that the provision of HCT is not a part of their routine practice, and pre-conceived notions make providers less willing to offer patients testing.

The two provider factors were identified as representing, ‘benefits of HIV testing’ and ‘comfort with providing HIV testing’. The factor scores in Table 3 signify the baseline attitudes of providers towards ED-based HCT. Provider factor scores show that ‘comfort with providing HIV testing’ has a greater negative influence on providers’ attitudes to offer HCT in the EDs.

### Determinants of testing attitudes

Among patients, a significant difference was observed in attitudes towards ‘counselling & testing’, ‘ED-based HCT’, and ‘stigma around HIV infection’. Patients identifying as ‘Coloured’ have a more positive attitude towards ‘counselling & testing’ than those identifying as ‘Africans’ (p = 0.005), and less positive attitude than those identifying as ‘White’ (p = 0.008). Patients who reported employment have a more positive attitude towards ‘ED-based HCT’ than those who didn’t (p = 0.045). Patients identifying as ‘White’ have a more positive attitude towards ‘stigma around HIV infection’ than those identifying as ‘Africans’ (p = 0.03). There were no significant differences in attitudes observed by patients’ age, gender and level of education, and providers’ age, gender, race, level of education, current position, and years of medical practice.

## Discussion

To conquer the HIV epidemic in South Africa, innovative testing venues are actively being sought. The ED is one such venue. While ED-based HCT has been extensively evaluated in North America and Europe, it remains relatively novel in this region [2, 8, 19, 32]. Although research supports that expanding HCT services to EDs has the potential to close critical coverage gaps, successful implementation of ED-based HCT programs depends significantly on the willingness and commitment of ED patients and providers to adopt the intervention. In this study we develop and present a refined survey instrument to evaluate attitudes towards ED-based HCT, which can allow for more precise and targeted interventions, as well as a more efficient and standardized assessment of attitudes towards HCT across contexts. Overall, results suggest that ED patients and providers have a neutral attitude to ED-based HCT. Factor analysis demonstrated patients reporting favorably on ‘counseling and consent’ and ‘ED-based testing’, but raised notable concerns regarding ‘confidentiality’ ‘stigma around HIV testing’, and ‘stigma around HIV infection’. Similarly, providers reported favorably on the ‘benefits of HIV testing’, but raised concerns around ‘comfort with providing HIV testing’.

Patient acceptance of ED-based HCT, in South Africa and elsewhere, is encouragingly high [1, 4–6, 24, 33, 34]. Our study revealed that 92% of patients expressed support for ED-based HCT, and 80% were willing to undergo testing, if available–findings which are consistent with other contexts [3, 5, 24, 33, 35]. Despite this reassuringly high acceptance combined with innovative measures undertaken by the government to reach all vulnerable, high-risk populations with HCT, an estimated 10% of HIV positive South Africans remain unaware of their status and 15% of all new infections globally continue to occur here [10]. In our study, patients of African race displayed significantly more negative attitude towards ‘counselling and testing’. In this setting, refusal of HCT services by this demographic may be explained by structural violence exercised over decades, encompassing institutional racism, environments with elevated burden of disease, and stigmatizing social norms preventing underserved populations from getting adequate healthcare [36, 37]. Wherein, individuals are asked to govern their own access to health care rather than relying on the state to provide necessary services, likely discouraging them from wanting to engage with the healthcare system. This further emphasizes the need to provide quality services for counselling and obtaining informed consent with transparency, especially as 80.8% of patients presenting to the ED were African. We also found, patients who were unemployed displayed a significantly more negative attitude towards ‘ED-based testing’, potentially stemming from a lack of awareness, misconceptions around HIV infection and transmission, and a lack of trust in the government [38]. Considering 62.5% of patients presenting to the ED reported being unemployed, they are another important demographic to capture.

Patients were concerned about confidentiality related to HCT in the ED. In a busy clinical environment with high patient volumes, where beds are separated only by curtains, ensuring confidentiality when testing and disclosing results remains a challenge. Moreover, misconceptions around HIV testing, such as the assumption that those agreeing to test are likely HIV positive, augment resistance to ED-based HCT. Challenges to maintaining confidentiality is inherent in an ED-based program design, where conducting HCT bedside is as much a problem as stepping away to a dedicated space [23, 26, 28, 39]. It is then imperative for facilities to continually sensitize their staff so as not to add to or reinforce any stigma constraining people’s testing decisions. Embedding HCT within routine clinical pathways can ensure the service is normalized, reducing resistance [40, 41].

In comparison to other studies evaluating challenges to implementing ED-based HCT in low-resource and high-prevalence regions, patients in our study strongly supported the provision for opt-in consent and counselling prior to testing. This is in contrast to the growing body of literature proposing an opt-out strategy eliminating the need for obtaining separate consent, as well as a flexible counselling program tailored to those who choose it [35, 42]. Although CDC guidelines state that risk reduction and prevention counselling should not be required as part of an HIV screening program, this recommendation must be viewed in context. In well-functioning networks, it may be assumed once a patient is diagnosed for HIV, they will be linked to care, and will receive appropriate counselling along with follow up care. However, in a setting such as an ED in a resource-poor context, ensuring linkage to care is still a challenge. Additionally, the historically unequal distribution of power and resources between racial groups in South Africa, giving rise to the idea of healthcare services being a commodity–available only to those who can afford it–has resulted in a lack of trust in the government [43]. This is exemplified in our data as patients agree that ‘the hospital already tests every patient for HIV without telling them about it’ and ‘if I have been to the hospital, and no one told me I had AIDS or HIV, then I am negative’. Therefore, while streamlining HCT into ED processes is beneficial, it should be approached with caution to avoid patients believing they could be tested without their knowledge.

Prior to this study there were no routine HCT efforts in these EDs or in the hospitals at large, despite South Africa’s nationally recommended provider-initiated HCT strategy in all health care settings as a standard component of medical care [44]. While 96% of providers believed all ED patients should know their HIV status, 52% believed HCT takes up too much of their time and interferes with their primary role, and 76% reported a shortage of staff and other resources, that would make implementing such a program a significant challenge. Frequently reported barriers to HCT, as well as other preventive programs in the ED, including domestic violence and alcohol screening in other settings, parallel the concerns noted by providers in our study [12, 27]. Several studies have explored the feasibility of a parallel model, wherein dedicated staff are hired to conduct HCT, as a routine part of clinical care in the ED [45]. A parallel model can alleviate the burden of providers taking on an additional role allowing them to focus on patients’ clinical complaints, also addressing concerns around shortage of staff. In line with this approach, 71% of patients and 89% of providers reported preferring an HIV counsellor for HCT, over a doctor or nurse, likely increasing acceptability. Requiring sustainable funding to hire and support dedicated staff is a drawback to consider, however, if suitably integrated into routine ED operations, ED-based HCT has been found to be persuasively cost-effective [46].

Our study also revealed a surprising amount of provider stigma towards people living with HIV, with almost 70% believing that HIV positive patients indulge in risky activities, and 20% believing that HIV positive patients present a threat to their health and thus have the right to refuse care. In the 1980s there was worldwide debate as to the ethical and moral obligations of healthcare workers to care for HIV positive patients [47]. Science organizations such as the World Medical Association, and professional bodies within South Africa and internationally have clearly worded statements that healthcare workers may not ethically refuse to treat a patient whose condition is within the physician’s current realm of competence, solely because the patient is seropositive [48]. The challenge however is breaking the barrier of stigma. To combat stigma in health facilities, interventions must focus on the individual, environmental, and policy levels, this means increasing awareness of what stigma is among healthcare workers, providing supplies and equipment necessary to practice universal precautions and prevent occupational transmission of HIV, and developing clear institutional guidance to stop discriminatory behavior [49].

Another commonly cited attitudinal barrier rendering providers generally unwilling to offer HCT is discomfort, stemming from the fear of upsetting or offending patients in an already vulnerable setting and the fear of contagion [27, 50]. This is supported by our results, wherein providers expressed discomfort in approaching patients for HCT, in disclosing the results of a positive test, and expressed a right to refuse care to HIV positive patients. Discomfort may be rooted in cultural stigma against individuals positive for HIV, wherein despite gains in knowledge a subset of physicians, across the globe, perceive no ethical difficulty in denying medical care to patients infected with HIV [51]. Fueling this notion, former President of South Africa, Thabo Mbeki and his administration established policy that denied access to treatment for individuals with HIV/AIDS. Despite international protections and a shift in policy in the last few decades, many providers continue to refuse treatment [52]. Interestingly, a majority of providers participating in our survey were nurses or providers who have been in practice for less than 1 to 4 years. Across low-, middle, and high-income settings discriminatory attitudes and unethical behaviours by residents and nursing staff towards HIV positive patients is well documented, underpinned by the perception of patient ignorance towards risk behaviours and a failure to link to care [53, 54]. Similarly, a positive attitude to, and increased comfort with HCT is associated with increased patient acceptance of HCT, as well as an increased sense of familiarity with, and confidence in conducting HCT for providers. Employing a phased approach to implementation, supplemented with regular education and training sessions for providers may address existing misconceptions and knowledge gaps that can lead to changes in attitudes and greater buy-in for the program.

Lastly, while this brief attitudes survey provides some insight to the organizational readiness for an ED-based HIV testing intervention; it only considers attitudes towards HIV–such as stigma, in addition to HIV knowledge, and attitudes towards testing. This may provide some pre-implementation opportunities to optimize patient and provider readiness for ED-based testing programs. Beyond provider concerns that testing would take too much time and the ability to deliver testing in a confidential manner, larger implementation concerns are not addressed. Other structured frameworks that are able to evaluate the broader implementation environment i.e., administrative resources, post-testing care etc. are required. Several such frameworks exist including the Theoretical Domains Framework (TDF), Modes of Diffusion in Service Organization (MDISO) and the Innovation Implementation Framework to name a few [55–57].

There are several limitations of the study to consider. Due to convenience sampling participants assembled for this study may be biased on multiple accounts: (1) those who were willing to complete the survey may be more likely to be supportive of an HCT program than those who declined, (2) patients enrolled in the study may have been easier to approach, spoke the same language as the study staff, had milder injuries, and presented at a time when patient volume was lower, and (3) a majority of providers surveyed were nurses, as physicians were generally fewer in number, but also centrally involved in all cases and hence less available. Thus, the study may under-represent true attitudes held by patients and providers in this context. Our study is unique in that it utilized exploratory factor analysis to better understand key determinants of attitudes towards ED-based HCT. To improve generalizability, it will be useful to see whether data from different samples show similar factor structures.

## Conclusion

Our study demonstrated ED patients and providers are generally supportive of ED-based HCT, though despite national policy in place since 2010, scale-up of HCT has remained inadequate. It is imperative patients and providers recognize HIV as an ongoing epidemic; it remains a leading cause of death and illness in South Africa. However, with a neutral attitude, high patient volumes, and disease severity of presenting patients, ED-based HCT is unlikely to result in successful implementation without behavioral interventions. A validated survey instrument can provide a standardized approach to understand and conquer existing barriers to HCT in an ED setting, across contexts.

## Supporting information

S1 Table Patient questionnaire (pre-validation). (DOCX)

S2 Table Provider questionnaire (pre-validation). (DOCX)

S3 Table Patient and provider factors with included attitudes questions. (DOCX)
