# Supplementary material for: Assessing attitudes to ED-based HIV testing: Development of a short-structured survey instrument
Source: PLoS One. 2021 May 27;16(5):e0252372. doi: 10.1371/journal.pone.0252372 (PMC8158958; doi:10.1371/journal.pone.0252372)
Supplement: S1 Table — (DOCX) [file pone.0252372.s001.docx]

**S1 Table: Patient Questionnaire (Pre-validation)**

| 1. Are you Male or Female? 2. Which of these Race Categories fit you? (You may check more than one if you identify with more than one group.) 3. What is your age? 4. What is your marital status? 5. What is your education level? 6. Are you employed? 7. Do you have a regular doctor? 8. Have you ever had an HIV test with your primary care physician? 9. Have you ever been tested for HIV somewhere other than your primary care clinic? 10. How long ago was your last HIV test? 11. Since the age of 18, how many times have you been tested for HIV? 12. Where were you tested? 13. What was the result of your last HIV test? 14. If you were offered an HIV test in this Emergency Clinic today, would you say yes? 15. Have you disclosed your HIV status to at least one person? If yes, to whom: 16. Are you on ARVs? 17. What is the reason for your emergency department visit? 18. Risk Behavior: Have you ever? - Are you sexually active? 19. Risk Behavior: Have you ever? - Had sex without a condom 20. Risk Behavior: Have you ever? - Had sex for drugs/money 21. Risk Behavior: Have you ever? - Had sex with a person with HIV or AIDS 22. Risk Behavior: Have you ever? - Had sex with a male who has had sex with a male 23. Risk Behavior: Have you ever? - Been a victim of sexual assault 24. Risk Behavior: Have you ever? - Had an unplanned pregnancy 25. Risk Behavior: Have you ever? - Had sex with an injection drug user 26. Risk Behavior: Have you ever? - Had sex while using drugs 27. Risk Behavior: Have you ever? - Injected drugs 28. Risk Behavior: Have you ever? - Had thoughts of suicide 29. Risk Behavior: Have you ever? - Drank too much alcohol 30. Risk Behavior: Have you ever? - Do you feel safe at home? 31. HIV Knowledge: Tell me if the following statements are true or false: - HIV is transmitted by having sex without a condom 32. HIV Knowledge: Tell me if the following statements are true or false: - HIV is transmitted by having sex with multiple partners 33. HIV Knowledge: Tell me if the following statements are true or false: - HIV is transmitted through injection intravenous drug use 34. HIV Knowledge: Tell me if the following statements are true or false: - HIV is transmitted by an HIV-infected pregnant woman infecting her baby 35. HIV Knowledge: Tell me if the following statements are true or false: - HIV can be prevented using condoms 36. HIV Knowledge: Tell me if the following statements are true or false: - HIV is caused by a virus 37. HIV Knowledge: Tell me if the following statements are true or false: - One can be HIV-positive and not have AIDS symptoms 38. HIV Knowledge: Tell me if the following statements are true or false: - HIV can cause death 39. HIV Knowledge: Tell me if the following statements are true or false: - HIV causes AIDS 40. For each statement below, tell us your opinion: - The A&E should offer HIV testing. 41. For each statement below, tell us your opinion: - I want to learn more about the HIV. 42. For each statement below, tell us your opinion: - I want to learn ways to avoid getting HIV. 43. For each statement below, tell us your opinion: - If offered HIV testing (in the A&E), I would get tested today. 44. For each statement below, tell us your opinion: - In order to be HIV tested, I would rather get my gums swabbed than my finger poked for a drop of blood. 45. For each statement below, tell us your opinion: - HIV testing should be free. 46. For each statement below, tell us your opinion: - I prefer to get my HIV results immediately (30 minutes-1 hour) rather than in 1-2 weeks. 47. For each statement below, tell us your opinion: - It doesn't matter who tells me my HIV result. 48. For each statement below, tell us your opinion: - The results of a negative HIV test can be disclosed where beds are separated only by curtains. 49. For each statement below, tell us your opinion: - The results of a positive HIV test can be disclosed where beds are separated only by curtains. 50. For each statement below, tell us your opinion: - I think that the hospital already tests every patient for HIV without telling them about it. 51. For each statement below, tell us your opinion: - If I have been in the hospital, and no one told me I had AIDS or HIV, then I am negative. 52. For each statement below, tell us your opinion: - The ED and hospital can test you for HIV without asking for your consent. 53. For each statement below, tell us your opinion: - You can catch AIDS or HIV by having your blood drawn in the hospital. 54. For each statement below, tell us your opinion: - The Point of Care HIV testing is not really confidential. 55. For each statement below, tell us your opinion: - My family would support me if I decided to be tested for HIV. 56. For each statement below, tell us your opinion: - I would not want anyone to know if I decided to be tested for HIV. 57. For each statement below, tell us your opinion: - Anyone who is tested for HIV is disgusting. 58. For each statement below, tell us your opinion: - I would be afraid to get an HIV test because people who test positive cannot get health insurance. 59. For each statement below, tell us your opinion: - People assume that everyone who is tested for HIV is infected with HIV. 60. For each statement below, tell us your opinion: - My parents would be upset if they knew I was planning to get tested for HIV. 61. For each statement below, tell us your opinion: - My friends would support my decision to get an HIV test. 62. For each statement below, tell us your opinion: - I am afraid that if I were tested for HIV, my name would go into public records. 63. For each statement below, tell us your opinion: - The Point of Care HIV tests give accurate results. 64. For each statement below, tell us your opinion: - It would be embarrassing to get tested for HIV. 65. For each statement below, tell us your opinion: - I would not consider getting an HIV test because I would be asked about things I have done that could get me in trouble. 66. For each statement below, tell us your opinion: - People would assume I have HIV if I decided to get tested. 67. For each statement below, tell us your opinion: - Anyone who is tested for HIV is smart. 68. For each statement below, tell us your opinion: - I trust the HIV test counselors and nurses to keep my information private and confidential. 69. For each statement below, tell us your opinion: - It would bother me if someone I know sees me getting an HIV test. 70. For each statement below, tell us your opinion: - My job would be in danger if my boss found out I was tested for HIV. 71. For each statement below, tell us your opinion: - Even if I test positive for HIV I may never get sick. 72. For each statement below, tell us your opinion: - Admitting that you should be tested for HIV means you have engaged in immoral behavior. 73. For each statement below, tell us your opinion: - Anyone tested for HIV is dirty. 74. For each statement below, tell us your opinion: - Patients should be provided with counseling prior to the offering of testing. 75. For each statement below, tell us your opinion: - Patients should be required to give consent prior to testing. 76. I am willing to pay this much to get an HIV test: ______ 77. The person who offers and HIV test should be a: (check all that apply) 78. The person who tells a patient their HIV test is positive should be a: (check all that apply) 79. The person who tells a patient their HIV test is negative should be a: (check all that apply) 80. I would NOT want to get an HIV test in the Emergency Clinic because: (mark all that apply) 81. Would you recommend a friend to get an HIV test in the Emergency Department? 82. If you were found to be HIV positive today where would you go for follow up or to start medications? 83. Any additional comments |
| --- |
