# Supplementary material for: Assessing attitudes to ED-based HIV testing: Development of a short-structured survey instrument
Source: PLoS One. 2021 May 27;16(5):e0252372. doi: 10.1371/journal.pone.0252372 (PMC8158958; doi:10.1371/journal.pone.0252372)
Supplement: S2 Table — (DOCX) [file pone.0252372.s002.docx]

**S2 Table: Provider Questionnaire (Pre-validation)**

| 1. Are you Male or Female: 2. Which of these Race Categories fit you? (You may check more than one if you identify with more than one group.) 3. What is your age? 4. What is your education level? 5. What is your current position at the hospital 6. Years at this hospital 7. Years of practice in your field 8. The Emergency Department should offer immunizations and/or immunization updates 9. The Emergency Department should offer HIV testing 10. Offering HIV testing will take too much time and will interfere with my job duties 11. I am afraid that if we ask patients about HIV testing, they will be offended or upset 12. The Emergency Department should offer HIV testing to high-risk patients only 13. The Emergency Department should offer HIV testing to ALL patients 14. I am comfortable disclosing the results of a positive HIV test to a patient 15. I am comfortable disclosing the results of a negative HIV test to a patient 16. I have adequate support staff for counseling and referral 17. The paperwork required for HIV testing is not too cumbersome 18. The following person should disclose the results of a positive HIV test to a patient 19. The following are barriers that would prevent me from providing HIV testing 20. If there are other barriers that would prevent you from providing HIV testing, please specify: 21. I believe most HIV+ patients acquired the virus through risky behavior. 22. I think HIV+ patients have engaged in risky activities despite knowing these risks. 23. I believe I have the right to refuse to treat HIV+ patients for the safety of other patients. 24. I think people would not get HIV if they had sex with fewer people. 25. HIV+ patients present a threat to my health. 26. HIV+ patients present a threat to the health of other patients. 27. I believe I have the right to refuse to treat HIV+ patients if other staff members are concerned about safety. 28. I would avoid conducting certain procedures on HIV+ patients. 29. I think if people act responsibly they will not contract HIV. 30. HIV+ patients tend to have numerous sexual partners. 31. I believe I have the right to refuse to treat HIV+ patients if I feel uncomfortable. 32. I would rather not come into physical contact with HIV+ patients. 33. I would want to wear two sets of gloves when examining HIV+ patients. 34. I believe I have the right to refuse to treat HIV+ patients to protect myself. 35. I would be comfortable working alongside another health care provider who has HIV. 36. I think many HIV+ patients likely have substance abuse problems. 37. I believe I have the right to refuse to treat HIV+ patients if I am concerned about legal liability. 38. I would rather see an HIV-negative patient than see an HIV+ patient with non-HIV-related concerns. 39. HIV+ patients should accept responsibility for acquiring the virus. 40. I worry about contracting HIV from HIV+ patients. 41. I often think HIV+ patients have caused their own health problems. 42. HIV+ patients make me uncomfortable. 43. I would be hesitant to send HIV+ patients to get blood work done due to my fear of others safety. 44. It is a little scary to think I have touched HIV+ patients. 45. I worry that universal precautions are not good enough to protect me from HIV+ patients. 46. I would feel uncomfortable knowing one of my colleagues is HIV+. 47. HIV+ patients who have acquired HIV through injection drug use are more at fault for contracting HIV than HIV+ patients who have acquired HIV through a blood transfusion. 48. I tend to think that HIV+ patients do not share the same values as me. 49. HIV+ patients who have acquired HIV through sex are more at fault for contracting HIV than HIV+ patients who have acquired HIV through a blood transfusion. 50. It would be hard to react calmly if a patient tells me he or she is HIV+. 51. It is important that everyone know their HIV status. 52. Emergency Department patients will benefit from knowledge of their HIV status. 53. What advice would you give to us when designing an ED based testing strategy for South Africa? |
| --- |
