# Supplementary material for: Assessing attitudes to ED-based HIV testing: Development of a short-structured survey instrument
Source: PLoS One. 2021 May 27;16(5):e0252372. doi: 10.1371/journal.pone.0252372 (PMC8158958; doi:10.1371/journal.pone.0252372)
Supplement: S3 Table — (DOCX) [file pone.0252372.s003.docx]

**S3 Table: Patient and Provider Factors with Included Attitudes Questions**

| **Patient Factors**   1. Confidentiality  - The results of a negative HIV test can be disclosed where beds are separated only by curtains. - The results of a positive HIV test can be disclosed where beds are separated only by curtains.  1. Counseling and consent  - Patients should be provided with counseling prior to the offering of testing. - Patients should be required to given consent prior to testing.  1. Openness to HIV knowledge  - I want to learn more about HIV. - I want to learn ways to avoid getting HIV.  1. Stigma around HIV testing  - The ED and hospital can test you for HIV without asking for your consent. - People assume that everyone who is tested for HIV is infected with HIV. - I trust the HIV testing counselors and nurses to keep my information private and confidential.  1. ED-based HIV testing  - The ED should offer HIV testing. - It doesn’t matter who tells me my HIV result. - I think that the hospital already tests every patient for HIV without telling them about it. - If I have been in the hospital, and no one else told me I had AIDS or HIV, then I am negative.  1. Social support  - My parents would be upset if they knew I was planning to get tested for HIV. - My friends would support my decision to get an HIV test.  1. Stigma around HIV infection  - I would not want anyone to know if I decided to test for HIV. - Anyone who is tested for HIV is disgusting. |
| --- |
| **Provider Factors**   1. Benefits of HIV testing  - The ED should offer HIV testing. - The ED should offer HIV testing to ALL patients. - The ED should offer HIV testing to high-risk patients only. - ED patients will benefit from knowledge of their HIV status. - Offering HIV testing will take too much time and will interfere with my job duties.  1. Comfort with providing HIV testing  - I am afraid that if we ask patients about HIV testing, they will be offended or upset. - I am comfortable disclosing the results of a positive HIV test to a patient. |
